# A projection‐domain deep learning approach for respiratory motion correction of myocardial perfusion imaging using a multi‐pinhole solid‐state SPECT

**DOI:** 10.1002/mp.70543

**Published:** 2026-07-22

**Authors:** Dylan J Malenfant, Terrence D Ruddy, R Glenn Wells

**Affiliations:** ^1^ Division of Cardiology Department of Medicine University of Ottawa Heart Institute Ottawa Ontario Canada; ^2^ Department of Physics Carleton University Ottawa Ontario Canada

**Keywords:** cardiac SPECT, deep learning, respiratory motion

## Abstract

**Background:**

Myocardial perfusion imagining (MPI) is a nuclear medicine technique used in the assessment of coronary artery disease. Differences in the perfusion of the myocardium at rest and stress and regions of perfusion defects can be indicators of various cardiac disease states. Respiratory motion (RM) can result in the degradation of image quality and the appearance of artificial regions of perfusion deficit. While RM correction is possible, many methods involve time‐consuming calculation or additional equipment for motion tracking. This has limited the clinical uptake of such methods. This work presents a deep learning model for data‐driven RM correction on dedicated cardiac SPECT scanners.

**Purpose:**

An AI‐based method for estimating RM from gated projection data for MPI SPECT using a dedicated pinhole scanner was developed. Accuracy of these estimates and impact on image characteristics were assessed.

**Methods:**

RM motion parameters were calculated for rest and stress MPI scans using a minimized root mean squared (RMS) alignment of reconstructed respiratory gates for 90 patients supplemented with 33 NCAT simulated patients. Using these parameters, an AI network was trained to estimate motion based on gated projection data prior to reconstruction. Accuracy of AI predictions relative to RMS data were assessed for clinical scans and down‐sampled data to simulate noisy acquisitions. Scans were reconstructed with AI motion correction (MC), RMS MC, and no MC. Anterior myocardial wall thickness and its contrast relative to the interior of the ventricle were calculated in each case.

**Results:**

AI motion predictions were accurate to within 1.86 ± 0.05 mm at clinical noise levels, increasing to 2.07 ± 0.07 mm at 1/8 signal. MC with AI was shown to provide a statistically significant increase anterior wall contrast and decrease measured wall thickness relative to no MC, with comparable results to RMS MC.

**Conclusion:**

Direct AI‐based MC parameter estimation using gated projection data was shown to be feasible in clinical settings. Corrections based on AI estimates showed improved contrast and reduced myocardial wall thickness when incorporated into image reconstruction.

## 1 INTRODUCTION

Nuclear medicine is a powerful tool for the diagnosis and assessment of coronary artery disease (CAD). Myocardial perfusion imaging (MPI) with single photon emission computed tomography (SPECT) is a commonly used non‐invasive means of CAD detection and risk stratification. MPI scan durations are on the order of minutes, which makes the images susceptible to degradation through patient motion. Respiratory motion (RM) is frequently present as scans are typically acquired with the patient free‐breathing. RM is predominantly in the craniocaudal direction,^1^ resulting in a loss in apparent tracer uptake in the superior and anterior myocardial walls and a perceived broadening of the myocardial walls. The magnitude of RM in stress MPI acquisitions was found to be more than 10 mm in one third of patients.^1^ Motion of this magnitude was found to result in visual changes to tracer uptake and was a significant cause of reduced quality in clinical MPI. In cases of extreme motion, repeat imaging may be required, leading to increased test duration and greater utilization of clinical resources.

Modern SPECT cameras acquire listmode data that can be processed after a scan is completed. By binning counts according to a respiratory signal, these scanners can produce respiratory‐gated images, limiting the impact of the motion. A respiratory signal can be obtained using equipment such as respiratory bellows^2^ or belt,^3^ or optical tracking of external markers.^1^, ^4^, ^5^ These methods, however, require additional equipment and setup time, presenting barriers to implementation. Data‐driven methods for respiratory gating have been investigated, such as using 500 ms intervals of the listmode data to estimate center of mass motion curves for the heart.^6^ Data‐driven methods present a more accessible means of gating but can also require the reconstruction of many image frames which is computationally intensive and time‐consuming. Correction by these methods typically involves the extraction of a respiratory signal from the projection‐domain data which can then be used to produce a gated study. Each gate is independently reconstructed, aligned in image space, and summed. This has been accomplished using a proprietary REGAT software used to create 16 respiratory gates.^7^ A similar method using a center‐of‐mass estimation in the projection domain has been attempted by using an initial ungated reconstruction of the heart to allow for the estimation of a volume of interest (VOI) where cardiac motion may occur.^8^ This VOI was then forward‐projected to create a mask and isolate cardiac counts in the projection domain. Axial motion observed in the projection domain was then used to provide a surrogate signal of RM.

Respiratory gating subdivides the detected counts among the gates leading to lower‐count images with increased noise. The increase in noise can be avoided by aligning the gates to a common reference point and then summing after reconstruction or by incorporating the relative motion for the gates into the reconstruction algorithm itself. The alignment vectors for each respiratory gate can be estimated using external markers with tracking equipment as described above, but this requires the assumption of direct correlation of internal cardiac motion and external motion in addition to the aforementioned barriers to implementation. Alternatively, alignment vectors can be computed by registering the gated images to a reference image. A simple method of registration is to assume rigid‐body motion with translation and rotation and minimize the sum of squared differences between gated images.^5^, ^6^, ^8^ These methods are computationally inefficient, requiring substantial time to reconstruct gated images and compute alignment vectors.

In recent years, deep learning has found many uses in medical imaging, particularly in nuclear medicine, including generation of attenuation maps based on magnetic resonance images^9^, ^10^, ^11^ or based on data from both photopeak and scatter windows in SPECT,^12^ image enhancement in low‐dose or short acquisition time SPECT,^13^, ^14^, ^15^ and for inter‐frame motion correction (MC) in dynamic cardiac positron emission tomography (PET).^16^ Once trained, deep learning networks provide rapid means of non‐linear calculations such as estimation RM.

In this paper, we have developed an automated tool for motion vector estimation by using a deep learning model to process scans directly from gated projection data. This method employs a data‐driven gating method and does not require intermediate image reconstruction. It provides an accessible and rapid method of motion estimation in MPI scans.

## 2 METHODS

### 2.1 Data acquisition

Patient data was acquired as part of a University of Ottawa Heart Institute Myocardial Blood Flow Registry. All patients provided written informed consent for their data to be included in this registry. Use of the data for this study was approved by the University of Ottawa Heart Institute Research Ethics Board.

Scans were acquired using a 1‐day rest/stress protocol with 99 m‐Tc‐tetrofosmin with a 1‐ to 2‐h interval between rest and stress imaging. Scans were acquired using the Discovery NM530c camera (GE Healthcare). An 11‐min dynamic scan was conducted beginning immediately prior to radiotracer injection. Analysis was conducted using the final 5 min of each scan. Rest scans were acquired for 90 consecutive patients with 89 of those patients undergoing stress imaging. All patients had an intermediate‐to‐high pretest probability of CAD. Their characteristics are described in Table 1. To simulate noisier acquisitions, listmode data were temporally down‐sampled to retain 1 in every 2 ms, 1 in 4 ms, and 1 in 8 ms.

**TABLE 1:** Patient demographics for studies used in this work.

Patient demographics	
Age (yrs)	69 ± 10
Sex (M/F)	65/25
BMI (kg/m^2^)	27.5 ± 3.8
Activity at rest (MBq)	339 ± 38
Activity at stress (MBq)	1058 ± 113

Additional high‐motion studies were simulated using the NCAT digital torso phantom.^17^ Three hearts of varying size were simulated, each with RM amplitude ranging from 10 to 20 mm in 1 mm steps, providing an additional 33 scans for analysis. NCAT scans were analytically forward projected with their associated attenuation maps to generate projection data. The projections contained the effects of attenuation and collimator response but did not include scatter. Counts in these data were scaled to match those found in patient scans, and random Poisson noise was added to each projection set. These data were included in the training set for the neural network, and no phantom data is included in the final analysis.

### 2.2 Respiratory gating

A data‐driven respiratory gating was performed based on the spatially‐dependent count rate observed with multipinhole collimation. The count rate was calculated in 100 ms intervals and smoothed using a 1‐s box filter to reduce noise and changes due to cardiac contraction. Amplitude gating was then performed based on the fluctuations of the count rate, corresponding to changes in cardiac position, to yield 10 evenly spaced respiratory gates. While these gates divide the RM evenly, the dwell time at each location is not the same. As a result, the number of counts in each gate decreases as the heart moves away from its equilibrium position. The listmode data were sorted into gated projections and the gates with the lowest number of counts were omitted from analysis as long as 95% of the total counts in the scan were retained. This yielded final projection datasets with 6 to 8 respiratory gates.

### 2.3 Rigid‐body motion estimation

Ground truth motion parameters to train the model were calculated in image space using a minimization of the root mean squared (RMS) difference between each respiratory gate and the reference gate, which was the gate with the most counts in projection space. Images corresponding to the gates were independently reconstructed using a maximization a priori expectation maximization (MAP‐EM) algorithm with 10 iterations using a noise‐suppressing Green's prior and an image voxel size of 0.4 cm. No attenuation correction was performed.

**FIGURE 1:**
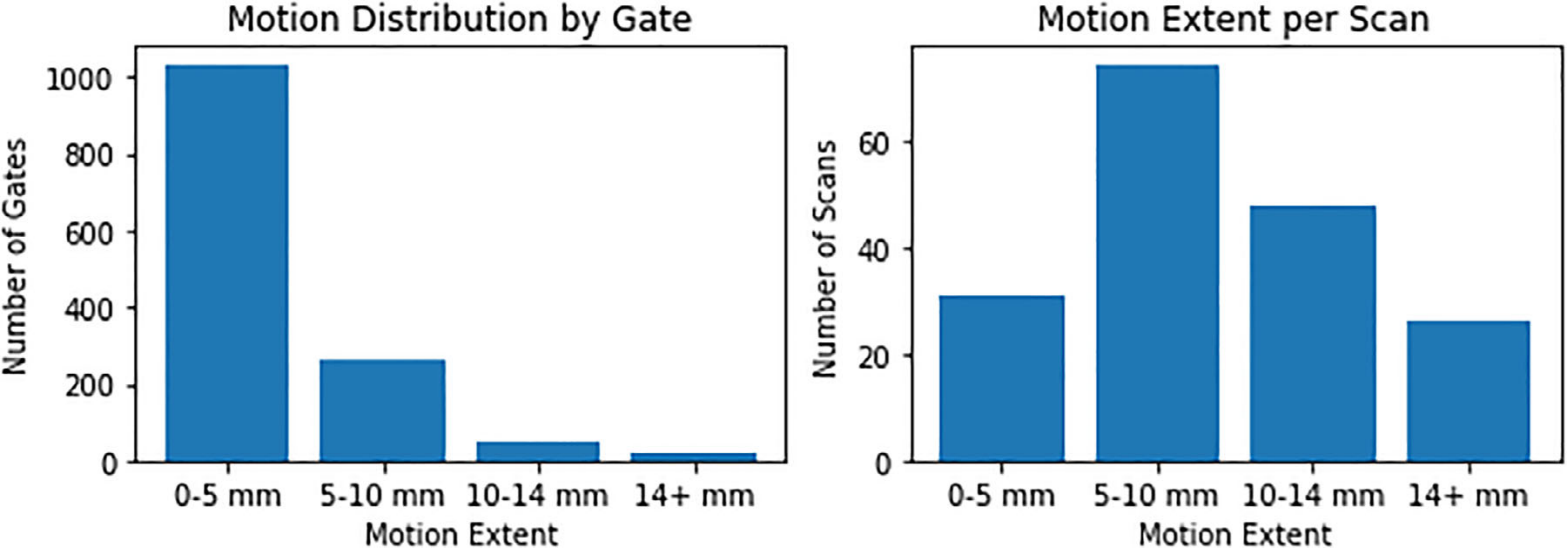
Distribution of motion per‐gate within scans and the distribution of total motion extents per scan. While 14.5% of scans show a total motion extent of 14 mm of more, this motion is only present in one gate in each of these scans, representing 1.2% of the individual image gates.

To reduce bias from extra‐cardiac activity, a spherical mask was centered on the heart in each gate. The total counts in the masked volumes were normalized across gates. Volumes were translated with three degrees of freedom to minimize the RMS difference in counts for each volume relative to the reference gate. The distribution of shifts relative to the reference gate for retained gates and the distribution of total motion extents amongst patient scans are shown in Figure 1.

### 2.4 Network architecture and training

**FIGURE 2:**
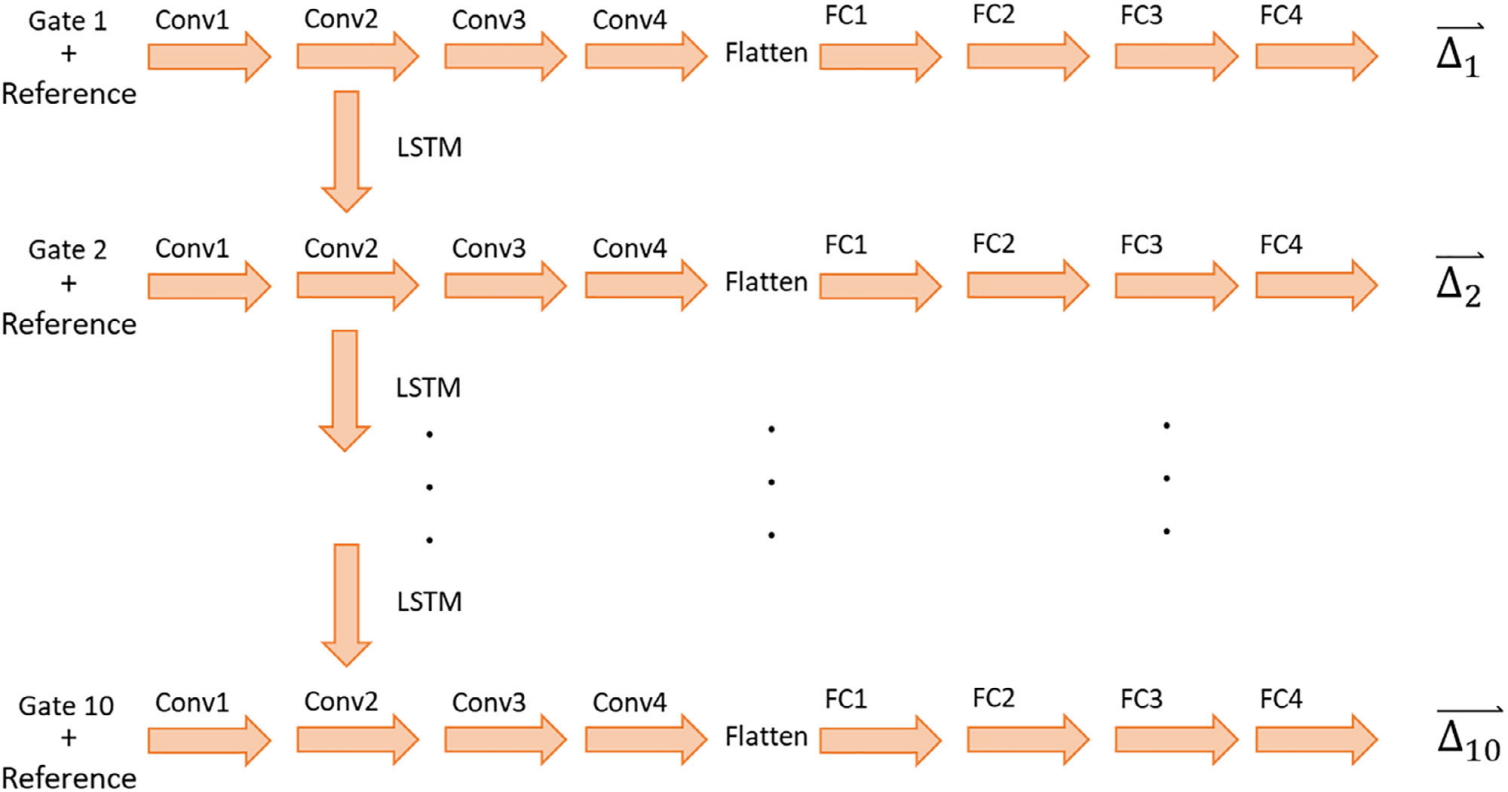
Diagram of the network architecture used for motion parameter estimation. Each gate is processed as a set of 19 projections concatenated with the reference gate to generate 3‐vector translational motion estimate, represented above as $\vec{\Delta_{i}}$ for the shift relative to reference for the *i*‐th gate. An LSTM cell connects to each following gate at the second convolutional layer. The LSTM passes information from each gate to the next, providing context between all ten input gates.

Machine‐learning derived motion vectors were computed using a convolutional neural network (CNN) including a long short‐term memory cell (LSTM). An LSTM cell acts to pass context data between time steps in sequence data using an additional set of trainable weights. In this case, a single LSTM cell was used such that image data from the previous gate could be included in the calculation of motion for each subsequent gate. Number and placement of the LSTM cell was determined by trial‐and‐error to minimize the training error in the network. This architecture is illustrated in Figure 2. For each respiratory gate, the 19 32×32 projections were concatenated with the projections corresponding to the reference gate to generate the network input. When gates were omitted due to low counts, the first and last retained gate were replicated to pad the array. Outputs from replicated gates were discarded. Each gate was passed through four convolutional layers (3×3 filters). A stride of 2 was employed for down sampling in place of a max pooling layer because pooling layers are local shift invariant,^18^ resulting in information loss around small shifts. A 2D convolutional LSTM was applied in the second convolutional layer. The data were then flattened, and four fully‐connected layers were used to generate a 10×3 set of motion parameters corresponding to the translational motion for each gate. This model was implemented using Keras^19^ and trained for 300 epochs with an Adam optimizer (β_1_ = 0.9, β_2_ = 0.999) with a learning rate of 0.0001. The network used a mean‐squared error loss (MSE) taking the image space motion estimates (RMS) as ground truth.

A ten‐fold cross‐validation scheme was employed with 90% of the patient data used for training and 10% withheld for testing, giving a training set of 161 patient scans and a test set of 18 scans. Ten instances of the network were trained, each using a different 90/10 split for training and test data, and final performance metrics are averaged across the ten instances. The errors presented are the standard deviation between instances. Phantom data were exclusively used as part of the training data and were not a part of the final analysis. Data were stratified by motion extent, with motion less than 5 mm considered small, motion between 5 and 10 mm as moderate, motion between 10 and 14 mm as high, and motion beyond 14 mm considered extreme. Each stratum was sampled such that 90% were included in the training set and 10% reserved for testing rather than randomly sampling the patient population. This was to prevent a network from being trained on a data set containing only patients with low motion extents, biasing the results of the training against high‐motion cases. Rest and stress data were treated as equivalent for the purposes of network training, separating the data exclusively based on the RMS‐estimated motion extent.

For each network, the average difference between network‐predicted (CNN) and RMS parameters was calculated for all gates. Bland‐Altman plots were used to test for bias in prediction in the craniocaudal direction and axial plane. To test the robustness of this method to noise, test data were down sampled and processed. Clinical data were down sampled to 1/2, 1/4, and 1/8 count levels. Networks were trained using down‐sampled training data to determine if this improved robustness with noise. A failure threshold was set at 3.5 mm, half of the camera resolution,^20^ where CNN estimation that agreed with RMS parameters within this margin was considered a success. Rates of success per gate were calculated for the stratified categories, with large and extreme motions aggregated into a single category.

### 2.5 Image analysis

Reconstruction of the final image used 50 iterations of the MAP‐EM algorithm with a Green's prior and 3D post‐reconstruction filtering using a Gaussian filter (12 mm FWHM). For motion compensation, the motion is incorporated as a translation using 3D linear interpolation within the forward and backward projectors. The data for each respiratory gate are weighted based on the amount of time spent to acquire each gate^21^ and are combined to update a single common image estimate. Three sets of images were generated: with motion parameters estimated by the true motion (RMS), with CNN‐generated motion parameters (CNN), and without correction (NoMC). For each scan, the heart was rotated to display in the vertical long axis. A profile was then drawn along the short axis through the anterior and inferior myocardial walls at the midpoint of the ventricle. The profile was then fit to minimize contributions from noise (Equation 1). The profile of the anterior wall was fit to a gaussian function with a constant background. In many images, the inferior wall was not discernable from the liver contributions, so the profile was truncated and the inferior wall was fit using an exponential function. The final function that data were fit to was

(1) $$I\left(x\right)=Ae^{{\left(\frac{x-B}{D}\right)}^{2}}+Ee^{Fx}+G$$

where A, B, D, E, F and G are fit parameters; I(x) is the counts in the profile from the image and *x* indexes the samples in the profile.

Using these profiles, the contrast of the anterior ventricular wall was calculated relative to the interior of the ventricle using

(2) $$C=\frac{I_{wall}-I_{int}}{I_{int}}\;$$

where $I_{wall}$ was the fitted peak intensity of the anterior ventricular wall, and $I_{int}$ was the fitted minimum intensity inside the ventricle. Wall thickness was calculated from the fit as the full‐width at half maximum of the anterior ventricular wall measured relative to the interior of the ventricle. Percentage change compared to NoMC, for both contrast and wall thickness, were plotted against total motion extent.

## 3 RESULTS

**FIGURE 3:**
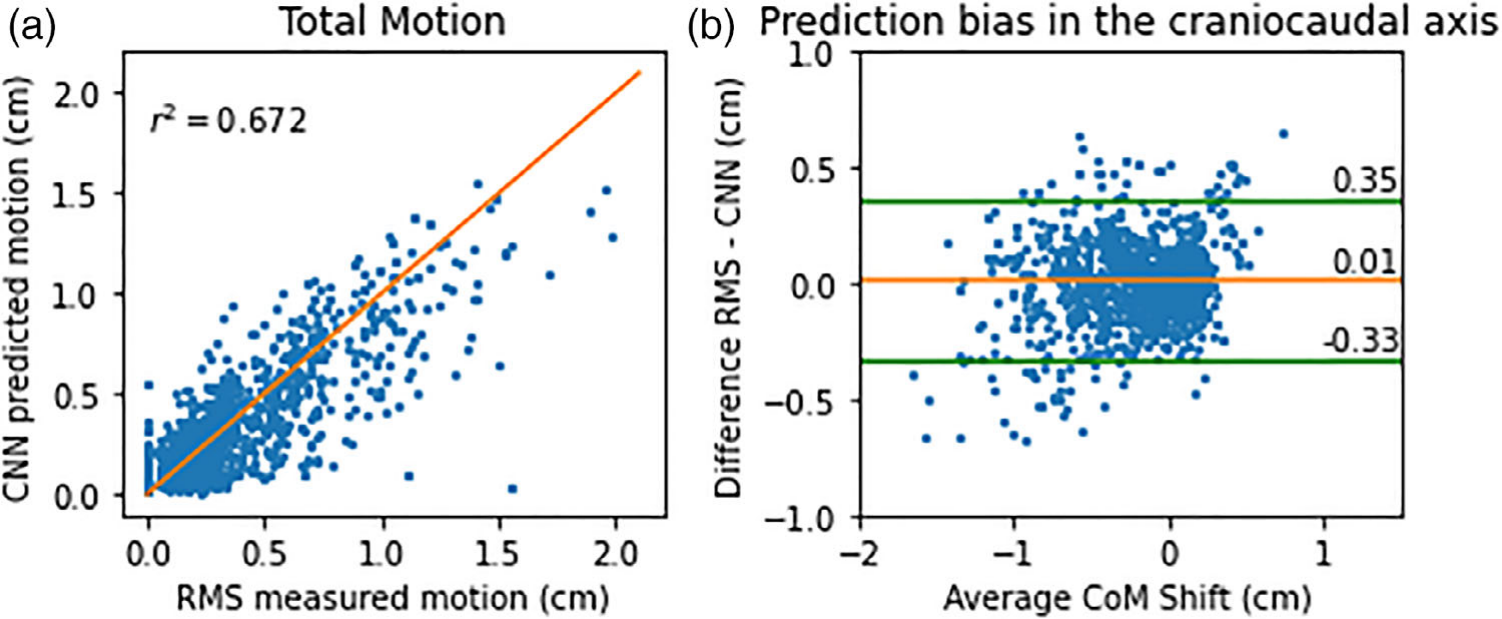
Estimated motion extent per gate compared to RMS measured motion (a), and a Bland‐Altman plot showing bias in axial motion predictions (b). Motion is strongly dominated by the cranio‐caudal direction, with 90% of patients showing sub‐pixel motion extent in the x‐ and y‐axes. These plots are dominated by noise, and so were not included.

**FIGURE 4:**
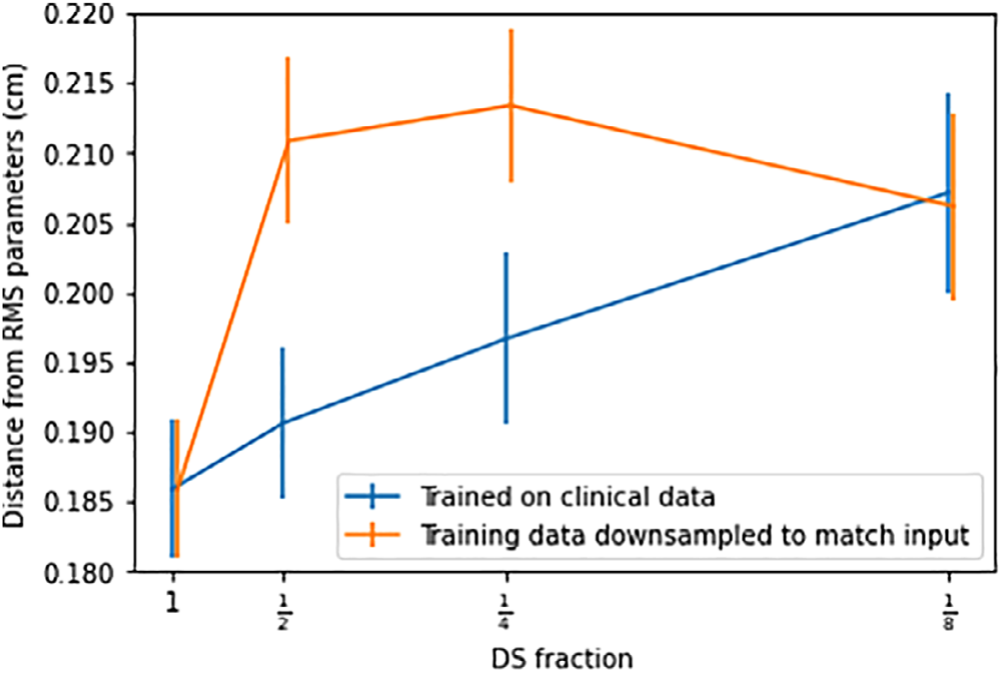
Error in parameter estimation averaged across 10 instances of the neural network. Down sample (DS) fraction gives the fraction of retained counts in the test images. Data sets are offset by 0.05 to avoid overlap. Data in blue analyzed using a network trained on clinical levels of noise, corresponding to DS = 1. In orange, individual networks were trained at each level of down sampling and used to analyze inputs of corresponding noise levels.

Network‐generated motion parameters found to agree with ground truth to within 1.86 ± 0.05 mm at clinical noise levels. Comparing RMS‐measured motion extent to CNN estimation for each gate yields a correlation coefficient of 0.672. No bias in these estimations is observed in the cranio‐caudal direction (Figure 3). Down‐sampling increases the difference between estimates to a maximum of 2.07 ± 0.07 mm at 1/8 clinical count levels (Figure 4). Motion estimates obtained by a network trained on clinical noise levels were more accurate than those obtained from a network trained on down‐sampled data at all noise levels.

Success rates for network predictions (Table 2) were based on gate‐by‐gate motion estimates. At all noise levels, small shifts were identified with over 91% accuracy. Network accuracy decreased with increased motion. For clinical levels of noise, moderate motion was correctly predicted with 74 ± 11% accuracy, dropping to 44 ± 20% for motion greater than 10 mm. Overall, an accuracy of 71 ± 21% was achieved across all patients at clinical noise levels.

**TABLE 2:** Success rates in estimating motion to within 3.5 mm.

	Relative motion			
Down sample fraction	Small (< 5 mm)	Moderate (5–10 mm)	Large (> 10 mm)	Total
Clinical	94.8 ± 2.8%	74 ± 11%	44 ± 20%	88.2 ± 3.2%
1/2	93.0 ± 3.0%	72 ± 11%	44 ± 23%	86.4 ± 3.3%
1/4	92.6 ± 4.6%	60.7 ± 8.8%	39 ± 21%	83.7 ± 4.0%
1/8	91.4 ± 3.9%	50 ± 13%	37 ± 25%	80.6 ± 4.1%

**FIGURE 5:**
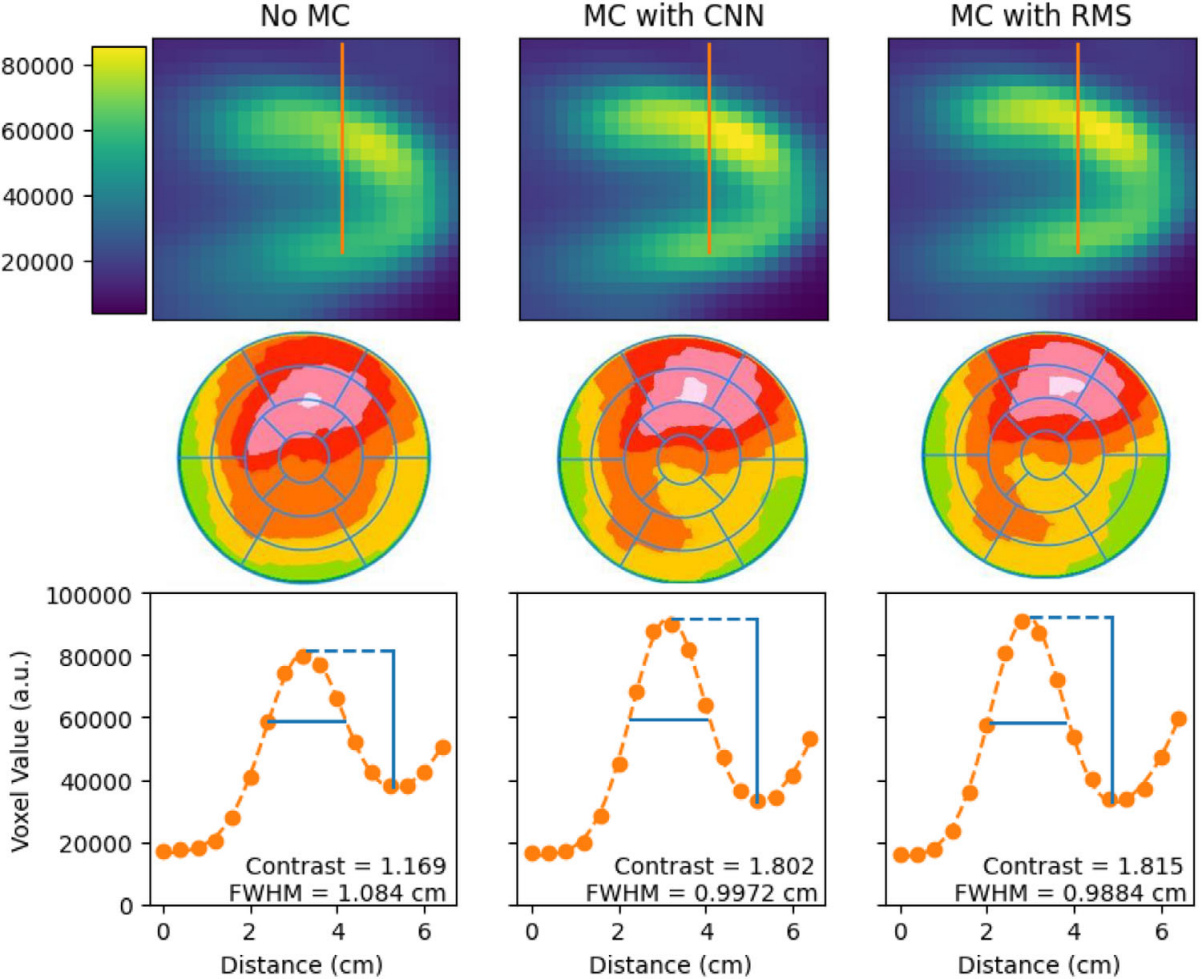
Representative profiles through the anterior wall of the heart. Voxel values are not normalized to reflect actual counts and are measured in arbitrary units (a.u.). The scan is shown with motion correction using a minimization of the RMS difference between gates (RMS) in image space and parameter estimation using the neural network in projection space (CNN) with the uncorrected scan (No MC) for comparison. Each image uses a common color scale and shows the profile location as an orange line. A 17‐segment perfusion map for each scan is also shown for each. For both methods of MC, a similar recovery in peak count in the myocardial wall is observed as well as a slight reduction in counts observed inside the ventricle (contrast indicated by vertical line). Likewise, the wall thickness (FWHM, horizontal line) is reduced with MC.

**FIGURE 6:**
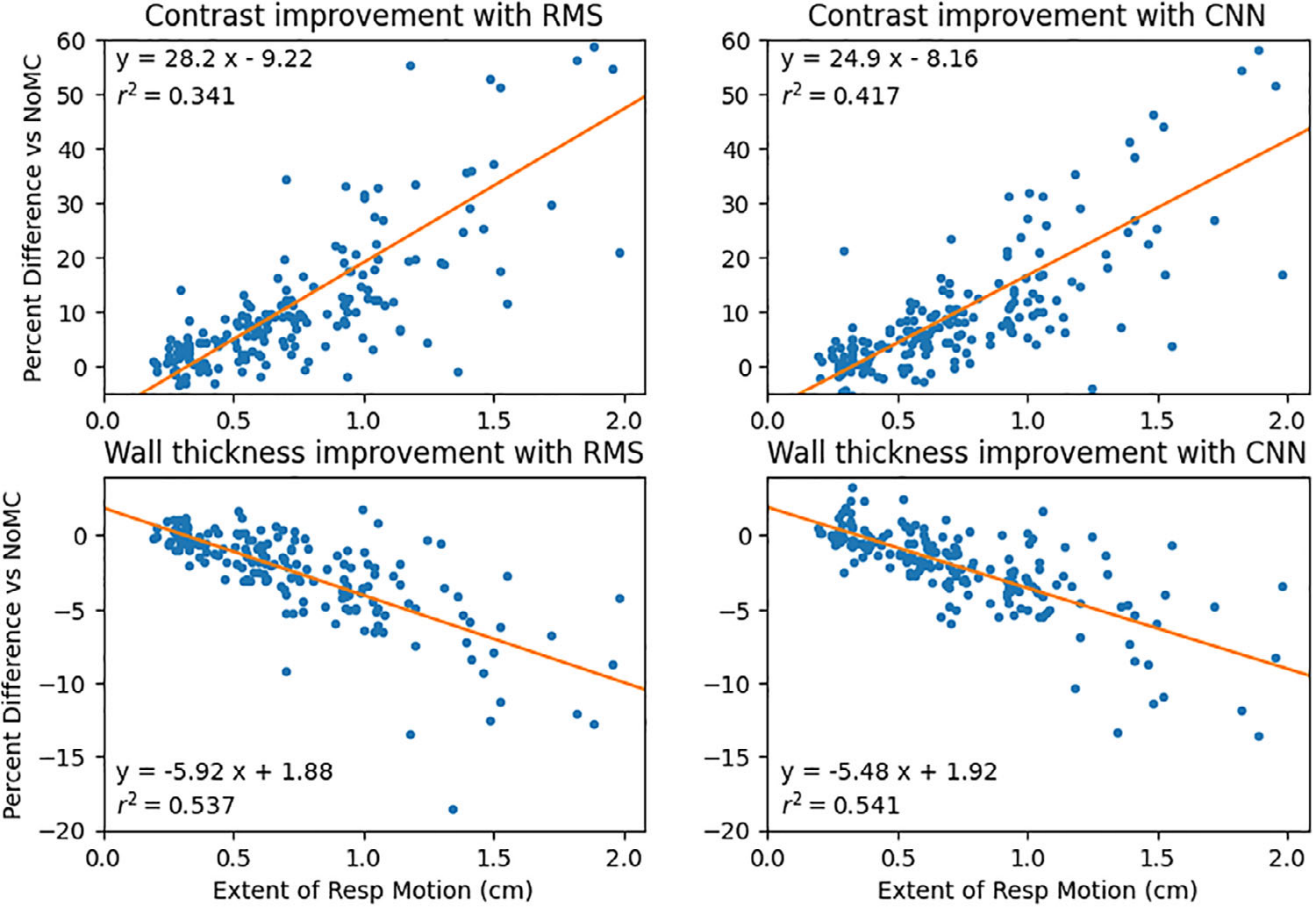
Contrast and wall‐thickness improvements for MC methods as a function of respiratory motion extent. While the degree of improvement is similar between methods, the difference was statistically significant (*p = 1.80e‐3* for change in contrast, *p = 8.15e‐6* for change in FWHM).

Images reconstructed using MC showed an improved anterior wall contrast relative to uncorrected images. Using parameters from the RMS minimization method, contrast increased by 29 ± 31% (*p = 6.67e‐10*) for patients with RM of greater than 10 mm relative to uncorrected images. An example scan is shown in Figure 5. MC using network‐generated parameters resulted in a contrast increase of 25 ± 24% (*p = 3.64e‐9*) in the same population relative to the uncorrected studies. Calculated anterior wall thickness decreased in scans with MC for patients with a motion extent greater than 10 mm. Wall thickness was measured as 5.9 ± 4.1% less when using the RMS minimization parameters (*p = 9.09e‐12*) and 5.1 ± 3.7% less with network‐generated parameters (*p = 3.46e‐11*). Improvements in both parameters was correlated with RM extent, shown in Figure 6, and average contrast and wall thickness values are given in Table 3.

**TABLE 3:** Anterior wall contrast and thickness with MC.

	No MC	MC with RMS	MC with CNN
Anterior wall contrast	1.42 ± 0.48	1.76 ± 0.63	1.73 ± 0.61
Wall thickness (cm)	1.14 ± 0.15	1.07 ± 0.11	1.08 ± 0.12

## 4 DISCUSSION

This work used deep learning to estimate motion parameters in respiratory‐gated SPECT MPI studies from projection data acquired using a dedicated cardiac multi‐pinhole collimated CZT scanner. This method bypasses the time‐consuming reconstruction required in many modern MC approaches. The data‐driven approach for gating and motion estimation ensured that this method can be adopted in clinical settings without an expert operator or additional equipment for respiratory tracking. This method showed an accuracy of 94.8 ± 2.8% in identifying images with motion of 5 mm or less relative to the reference gate. These gates represent approximately 75% of this data set.

The use of a neural network allows parameter estimation to occur with a respiratory‐gated set of projections from a patient scan without the need for reconstruction, taking only a few seconds on a standard clinic workstation. In comparison, reconstruction of all respiratory gates requires multiple minutes. Iterative rigid‐body approaches have shown success in the past,^5^, ^6^, ^8^ however, these methods add computational time on the order of minutes as well prior to a final motion‐compensated reconstruction. Because high levels of RM are only observed in roughly 1 in 3 patients, this addition of several minutes of processing is often not clinically relevant and so is often only applied when motion artefacts are recognized in the scan by a clinician. By using this AI approach, a motion estimate is provided immediately. In cases where motion is high, motion compensation can be performed with all reasonable expectation of clinical benefit. When motion is estimated to be low, no action needs be taken, as the network shows high accuracy in identifying low motion scans. In addition, without a means for motion estimation, manual alignment of gates requires direct input from an expert operator with the amount of time spent being operator dependent.

Using this method, such resources can be reserved for only cases where the correction is known to be necessary. Increased noise was observed to decrease motion estimate accuracy. Scans down‐sampled to 1/8 clinical signal showed an error of 2.07 ± 0.07 mm. In comparison, an error of 1.86 ± 0.05 mm was observed with no down‐sampling. At 1/8 clinical signal level, motion under 5 mm is identified with an accuracy of 91.4 ± 3.9%, decreased from 94.8 ± 2.8%. In motion ranges between 5 and 10 mm, down‐sampling resulted in a decrease in accuracy from 75 ± 11% to 50 ± 13%. With patients exhibiting more than 10 mm of motion, accuracy decreased from 44 ± 20% to 39 ± 25%.

Total motion extent is a strong predictor of accuracy of the neural network's motion estimate. Success rates for parameter estimation within 3.5 mm decreased from 94.8 ± 2.8% for gates with less than 5 mm of motion to 75 ± 11% for gates exhibiting motion between 5 and 10 mm, and to a rate of 44 ± 20% for gates with motion greater than 10 mm. This may be due to an inherent bias in the training data. While 41% of scans showed motion extents greater than 10 mm, only 5.1% of all gates show motion beyond 10 mm. This underrepresentation may lead to chronic underestimation of extreme motion. Phantom data with simulated RM was included in the training set to offset this effect. During the development of this network, it was found that error averaged across a 20‐patient test set was reduced from 1.78 ± 1.18 mm to 1.64 ± 1.03 mm, a decrease of 7%, when including phantom data in the training set. This set contained 154 retained gates. This effect was more prominent in gates with 10 mm or more of motion, where motion error was reduced from 3.49 ± 1.39 mm to 2.55 ± 1.03 mm, a decrease of 36%, across the 10 retained gates that met this motion threshold. It may be possible to train a MC network exclusively on a high‐motion population. Because the heart remains near to equilibrium for most gates even in a high‐motion scan, such a network should not be impacted with low‐ to moderate‐motion patients.

Large standard deviations are observed in the average contrast and wall thickness change after motion compensation. This is due to the impact of the shape of motion on blurring in addition to the range of motion. When motion is sharp, with most of the counts acquired in a single position, count smearing is relatively limited. A smooth motion profile over an equivalent range can result in a more significant loss of contrast and increase to observed wall thickness. As a result, some scans show little improvement after MC even after limiting the population to those with a significant motion extent.

Images reconstructed with network‐estimated motion parameters show a statistically significant increase in contrast and decrease in measured anterior wall thickness compared to uncorrected images. This indicates that estimated parameters reduce the blurring effects of RM despite some error in the accuracy of the motion displacement vectors. Improvements due to MC are statistically significant for both the true motion and CNN estimates. RMS parameters provide a statistically significant improvement over network‐estimated parameters. However, the difference between MC with RMS estimates and CNN motion estimate images is small, with a 4% increase in contrast and < 1% reduction in measured wall thickness using RMS estimates over CNN. Image reconstruction has not been optimized with respect to motion compensation, and further improvements to the FWHM and contrast may be possible. The methods used in this paper reflect the reconstruction methods used in current clinical operations.

This model is limited to translational motion. RM has a rotational component, with the apex pivoting about the base during respiration.^22^ However, this rotation is small.^1^ This model could be expanded to include rotations without significant restructuring. However, the impact of this rotational motion is only apparent in scans with extreme motion. This would only impact a small number of scans, and it would only be observable in a small number of low‐count gates. Few scans in this training set would show any effect, making integration of rotation into the present model impractical.

In this study, ground truth was obtained by minimizing the L_2_ norm between gated images and a reference‐gate image. For clinical data, actual motion data are unknown. Motion estimates were manually reviewed for overt errors, but residual errors may have an impact on the accuracy of the trained network. Increasing the size of the training set would reduce the impact of these potential errors in the final network.

Training data were dynamic scans which were acquired 6–11 min after tracer injection. Typical clinical images are acquired with a 30–45 min delay between injection and imaging. Immediately after injection, there is a higher likelihood of extra‐cardiac uptake of tracer which can interfere with evaluation of the myocardium.^23^ The difference in radiotracer distribution may affect the performance of the network when applied to typical MPI scans. Further evaluation is needed to assess performance with these images.

## 5 CONCLUSIONS

This work demonstrates a method for rapid RM estimation from respiratory‐gated projection data acquired on a multi‐head pinhole camera using a data‐driven gating scheme. This method provides improved contrast in the reconstructed image and reduces blurring in the anterior myocardial wall to an extent comparable to image‐space motion estimation methods and without the need for an expert user.

## CONFLICT OF INTEREST STATEMENT

RGW and TDR received research support from GE Healthcare.
